# A Survey of Statistical Methods for Microbiome Data Analysis

**DOI:** 10.3389/fams.2022.884810

**Published:** 2022-06-13

**Authors:** Kevin C. Lutz, Shuang Jiang, Michael L. Neugent, Nicole J. De Nisco, Xiaowei Zhan, Qiwei Li

**Affiliations:** 1 Department of Mathematical Sciences, The University of Texas at Dallas, Richardson, TX, United States,; 2 Department of Statistical Science, Southern Methodist University, Dallas, TX, United States,; 3 Department of Population and Data Sciences, The University of Texas Southwestern Medical Center, Dallas, TX, United States,; 4 Department of Biological Sciences, The University of Texas at Dallas, Richardson, TX, United States

**Keywords:** microbiome data, metagenomics data, differential abundance analysis, integrative analysis, network analysis

## Abstract

In the last decade, numerous statistical methods have been developed for analyzing microbiome data generated from high-throughput next-generation sequencing technology. Microbiome data are typically characterized by zero inflation, overdispersion, high dimensionality, and sample heterogeneity. Three popular areas of interest in microbiome research requiring statistical methods that can account for the characterizations of microbiome data include detecting differentially abundant taxa across phenotype groups, identifying associations between the microbiome and covariates, and constructing microbiome networks to characterize ecological associations of microbes. These three areas are referred to as differential abundance analysis, integrative analysis, and network analysis, respectively. In this review, we highlight available statistical methods for differential abundance analysis, integrative analysis, and network analysis that have greatly advanced microbiome research. In addition, we discuss each method’s motivation, modeling framework, and application.

## 1. INTRODUCTION

Bacteria, viruses, fungi, and other microscopic living things are referred to as microorganisms or microbes. The term *microbiome* describes the collective genomes of the microorganisms or the microorganisms themselves [1]. The human microbiome plays a vital role in controlling vital functions in the body such as immune system development, protection against pathogens, and modulation of the central nervous system [2]. The microbiome is dynamic and changes with factors such as diet or the use of antibiotics [3]. Changes in the microbiome may affect host health and cause disease [2, 4]. In the last decade, many advances in sequencing technology and statistical methodology have made it possible to study and quantify the microbiome.

In quantitative microbiome research, there are three popular areas of interest that seek to detect and quantify (i) differentially abundant taxa across phenotype groups, (ii) associations between taxonomies and covariates, and (iii) associations between taxa in the whole microbiome network. These three areas are referred to as differential abundance analysis, integrative analysis, and network analysis, respectively. Many useful methods have been developed to perform these downstream analyses while taking multiple issues into consideration that arise from differences in sequencing technology such as 16S ribosomal RNA sequencing (16S rRNA) or metagenomic shotgun sequencing (MSS) [5], technical issues of sequencing technology [6], the complex nature of sequencing count data [7], and choice of data normalization techniques [8].

16S rRNA and MSS are commonly used high-throughput sequencing technologies that generate raw count data for microbiome statistical analysis. Both technologies have their advantages and disadvantages. In 16S rRNA, the 16S ribosomal gene sequence is useful for the identification and classification of bacteria and archaea [9] because it is conservative as well as found in most microbes [10] and contains multiple sequences of the gene within a single microbe [11]. 16S rRNA is a relatively short sequence in the bacterial genome. Their sequences can be clustered as operational taxonomic units (OTUs) or amplicon specific variants, which better classify bacteria at the phyla and genera levels but is less precise at the species level. Further, 16S rRNA has available reference genomes and pipelines to perform data analysis such as DADA2, Mothur, and QIIME [12–15]. In contrast, MSS targets entire genomes with greater resolution giving it the capability to efficiently classify bacteria at the species level as well as describe microbial communities and their functional differences [16]. MSS identifies far more species per read than 16S rRNA and is more advanced because it can also identify viruses, fungi, and protozoa [12]. As a result, MSS is more costly per sample than 16S rRNA and so sample sizes tend to be smaller in studies with MSS data [17]. Of course, these are not the only existing sequencing methods. RNA-Seq, ChIP-Seq, and MeDIP-Seq are some of the many sequencing technologies that are also available.

Microbiome count data have characteristics that pose numerous challenges to methodology such as zero inflation, overdispersion, high dimensionality, and sample heterogeneity [18, 19]. Further, when count data are transformed to compositional data (i.e., total sum scaling), the counts in each sample are only relative to each taxon and do not necessarily reflect absolute abundance [20] due to variable sequencing depth across samples [5]. Zero inflation is common where possibly up to 90% of all counts are zeros [20]. Further, MSS count data are typically much more sparse than 16S rRNA data [5]. Some of the zeros are true zeros and others are false zeros. False zeros result from technical variability and limitations in sequencing depth when taxa with low abundance are completely missed at random [8, 16]. Quality of DNA preparations such as inconsistencies in the DNA extraction or how samples are handled can also contribute to technical variability [16, 18]. Library size is the total number of reads per sample (i.e., the sum of all the counts in a sample). Different library sizes (i.e., sample heterogeneity) result as a consequence of technical variability, which make it difficult to compare the samples. Samples with greater library size could contain higher reads for non-differentially abundant features, which would lead to the spurious conclusion that those features are differentially abundant [8]. Batch effects are problematic and may lead to spurious conclusions especially with MSS data, which is generated over multiple sequencing runs [18]. Furthermore, biological, technical, and computational factors are probable sources of batch effects [21]. Normalization alone does not fully correct for batch effects [22]. Many statistical methods are available that correct for batch effects including linear mixed models *via* the LIMMA package in R [23], metagenomeSeq in the Bioconductor software for users in R [24], Bayesian Dirichlet-multinomial regression meta-analysis (BDMMA) [25], surrogate variable analysis (SVA) [26], and remove unwanted variation (RUV2 and RUV4) [27]. Other methods that help to remove batch effects include batch mean centering (BMC) [28], ComBat [29], or its extension ComBat-seq [22], removeBatchEffect [23], FAbatch [30], RUVIII [31], percentile normalization [32], and singular value decomposition (SVD) [33]. Assumptions such as whether or not the batch effect is known or if the design is balanced must be considered when selecting an appropriate method to correct for batch effects. Wang and LêCao [21] provide a detailed decision tree that is helpful for identifying appropriate statistical methods that correct for batch effects.

In this paper, we first briefly describe the data one would typically encounter in microbiome data analysis and introduce their notations in Table 1. The sources referenced in this paper vary in their data notations and so we present notations in a consistent manner throughout this paper. Then, we discuss available methods for differential abundance analysis, integrative analysis, and network analysis. Specifically, we introduce the methods, applications, motivations, normalization techniques, models, statistical tests, and provide a brief discussion. Table 2 provides the classes and methods of statistical analyses discussed in this paper.

## 2. DIFFERENTIAL ABUNDANCE ANALYSIS

Microbial dysbiosis, or microbial imbalances, is related to disease. Microbiota have been implicated in the development of numerous diseases such as colorectal cancer [34], type 2 diabetes [35], liver cirrhosis [36], and inflammatory bowel disease [37]. The method of detecting differentially abundant taxa across phenotype groups is known as differential abundance analysis. Identifying differentially abundant taxa will help to understand the relationship between the symbiotic organism and human health as well as identify microbial biomarkers for disease screening. We discuss multiple methods for differential abundance analysis in this section including edgeR [38], metagenomeSeq [24], DESeq2 [39], analysis of compositions of microbiomes or ANCOM [40], a zero-inflated beta model or ZIBSeq [5], a zero-inflated generalized Dirichlet-multinomial model or ZIGDM [6], and count regression for correlated observations with a beta-binomial model or corncob [41]. The summary of these methods are found in Table 3 and their implementations for users in R are in Table 4.

The common biological motivation of each method is to determine if any particular features of $Y_{n\times p}$ are significantly different with respect to phenotype $z_{n\times 1}$ in a high-dimensional setting where the number of features is much greater than the number of samples (i.e., p≫n). Additionally, each method has its own statistical motivations. edgeR was motivated by the need to separate biological and technical variability in order to reduce bias when testing for significant phenotypic differences attributed to abundances of RNA-Seq data. Sparsity (i.e., zero inflation) is a common characteristic of MSS count data, which is one motivational factor for metagenomeSeq, ZIBSeq, and ZIGDM. For example, a particular bacterial species may be present in a small percentage of samples for both biological and technical reasons. Biologically, this particular bacterial species may be found in only a small percentage of samples. Limitations in technology such as sequencing depth may miss a particular bacterial species with low abundance completely at random. As a result, the number of zero counts becomes inflated. DESeq2 wanted a model that can account for the presence of outliers and small replicate sizes while producing interpretable results. Other methods including ZIBSeq, ANCOM, ZIGDM, and corncob were motivated by the need for models that can also account for the compositional nature of the count data. ZIGDM was further motivated by the need to account for correlation structure and dispersion patterns amongst features.

Normalization is a transformation needed for robust analysis that accounts for the challenges of microbiome data and technical variability of sequencing technology [8, 16, 18, 20]. Measurable, informative, and direct comparisons of samples are only possible after normalization [8]. Some methods for normalization are based on either sample-specific scaling of the raw counts or replacing the raw counts with normalized counts [16]. Popular normalization methods include but are not limited to total sum scaling (TSS), cumulative sum scaling (CSS), variance stabilizing transformation (VST), relative log expression (RLE), Aitchison’s centered log-ratio (CLR) or log ratio (ALR) of compositions, trimmed mean of M-values (TMM), and upper-quartile (Q75). The default normalization methods for edgeR, metagenomeSeq, DESeq2, ANCOM, and ZIBSeq are TMM, CSS, RLE, ALR, and TSS, respectively. Further, TMM, RLE, Q75, and TSS can be applied by both edgeR and DESeq2. Both ZIGDM and corncob apply model-based normalization. Model-based normalization estimates the normalized abundances *via* a distribution parameter rather than using a separate normalization step. The choice of normalization method produces more precise and biologically interpretable results when it is chosen appropriately. For instance, the CSS normalization technique used in metagenomeSeq scales the data with cumulative count sums up to a certain quantile. Further, CSS was shown to produce optimal model performance particularly for MSS count data. CSS is also helpful when there is zero inflation in the count data. Zero counts do not imply the nonexistence of a feature but could be the result of undersampling. On the other hand, TMM in edgeR helped to minimize the false discovery rate (FDR). Additionally, TMM attempts to trim away unwanted undersampling and oversampling effects in the log-fold changes. Normalization in edgeR was later updated to account for outliers by applying weights to the normalized counts. Similarly, DESeq2 accounts for outliers in the count data by taking advantage of the median-of-ratios or RLE normalization techniques, which could result in a more robust model. ANCOM normalizes the data *via* ALR to map the data from the simplex S to R, which allows for the use of classical tests such as ANOVA or Kruskal-Wallis to detect differential abundance. Normalizing the counts using TSS offers a way to model the compositional data directly as is done in ZIBSeq.

The microbiome data are assumed to follow a particular probabilistic model or distribution, which accounts for the noise in the count data. The probability density functions (pdf) or probability mass functions (pmf) and additional information for the parameters of each of the models are listed in Table 5. In general, the count data are sampled from

(1) $$y_{ij}|\cdot \sim ℳ(\cdot )$$

where ℳ(⋅) is a probabilistic model that depends on either the normalized or non-normalized abundances as well as other parameters such as mean or dispersion when applicable. The models discussed in this section choose candidates for ℳ to deal with at least one of the characterizations of count data. The negative binomial distribution (NB) is the candidate for ℳ for both edgeR and DESeq2 and can be generalized as $y_{ij}|\cdot \sim NB\left(\lambda_{ij},\phi_{j}\right)$ where $\lambda_{ij}$ is the mean and $\phi_{j}$ is the feature-specific dispersion parameter. The mean $\lambda_{ij}$ is parameterized as the product of a normalization factor $s_{i}$ and a parameter related to the count data $\mu_{ij}$, which is expressed as $\lambda_{ij}=s_{i}\mu_{ij}$. The choices for these two parameters for edgeR and DESeq2 are provided in Table 5. The variance of $y_{ij}$ is $\lambda_{ij}+\lambda_{ij}^{2}/\phi_{j}$. The variance increases as $\phi_{j}$ tends toward small values, which accounts for overdispersion in the count data [19]. However, the NB does not account for zero inflation. Next, a generalized linear model (GLM) using a log-link to model the mean abundance of feature j in sample i is fitted *via* maximum-likelihood estimation. The GLM can be written as $log\;\left(\mu_{ij}\right)=\beta_{j.}{\left(1,z_{i},x_{i.}\right)}^{⊤}$ where $\beta_{j.}=\left(\beta_{j0},\beta_{j1},\ldots ,\beta_{j,k+2}\right)$ are the regression coefficients for the intercept, phenotype, and covariates, respectively. Testing for differential abundance here is the equivalent of testing $H_{0}:\beta_{j1}=0$. edgeR uses the modified Fisher’s exact test by [50] where NB replaces the hypergeometric distribution; whereas, DESeq2 used the Wald test. Originally, edgeR was designed for only a binary phenotype but has since been updated to handle multiple groups.

Zero-inflated models help to significantly reduce the bias of sparse count data resulting from the undersampling effect of limited sequencing depth. The zero-inflated model is a mixture distribution with a continuous component and spike-mass at zero. An attractive feature of the zero-inflated model is the ability to estimate the probability that a zero count is a true zero (i.e., true absence of feature j) or false zero (i.e., undersampling) by the use of a discrete spike-mass at zero [51]. Zero-inflated models are employed by metagenomeSeq, ZIBSeq, and the ZIGDM. Generally, ℳ as a zero-inflated model can be expressed as $y_{ij}|\cdot \sim \pi_{i}I_{0}\left(y_{ij}=0\right)+\left(1-\pi_{i}\right)ℳ(\cdot )$. An equivalent way to model $y_{ij}|\cdot$ is by the use of a latent binary variable $r_{ij}$ such that

(2) $$y_{ij}\left\{\begin{array}{ll} =0 & \text{when}r_{ij}=1\\ \sim ℳ(\cdot ) & \text{when}r_{ij}=0 \end{array}\right.$$

where $\pi_{i}$ is the probability that $y_{ij}$ is zero [24] and $r_{ij}$ is sampled from a Bernoulli distribution with parameter $\pi_{i}$ [19]. A discrete spike assumes $y_{ij}=0$ with positive probability [51]; whereas, a continuous spike assumes $y_{ij}=0$ with zero probability [52]. The candidates for a zero-inflated ℳ are Gaussian or log-Gaussian, beta, and generalized Dirichlet-multinomial for metagenomeSeq, ZIBSeq, and the ZIGDM, respectively. Specifically, metagenomeSeq models the log_2_ continuity-corrected count data as ${\overset{˘}{y}}_{ij}={log}_{2}\;\left(y_{ij}+1\right)$ along with the CSS-normalized scaling factor $s_{i}$ for two population groups. Pseudo counts are created by adding a positive value to each $y_{ij}$ to avoid taking a logarithm at zero. The mean model here is written as $\mu_{ij}|\cdot =\beta_{j}\cdot {\left[1,z_{i},x_{i},{log}_{2}\;\left(C_{i}+1\right)\right]}^{⊤}$ where $\beta_{j.}=\left(\beta_{j0},\beta_{j1},\ldots ,\beta_{j,k+3}\right)$ are the regression coefficients for the intercept, phenotype, and covariates. The term ${log}_{2}\;\left(C_{i}+1\right)$ is the main contribution of metagenomeSeq, which includes the CSS-normalized value, $C_{i}$, that helps to remove bias due to extremely large counts in any sample. The expectation-maximization (EM) algorithm is used to fit the model and estimate the parameters. Testing for differential abundance here is the equivalent of testing $H_{0}:\beta_{j1}=0$. Then, metagenomeSeq computes *q*-values for multiple testing from a modified t-statistic calculated *via* Empirical Bayes. One issue here is that FDR increases when either sample size or effect size increases [20].

ZIBSeq models the relative abundances *via* a parametrized beta distribution [53] where ${\tilde{y}}_{ij}\sim Beta\;\left(\mu_{ij},\phi_{ij}\right)$ has mean $\mu_{ij}$, precision $\phi_{ij}$, and variance $\mu_{ij}\left(1-\mu_{ij}\right)/\left(\phi_{ij}+1\right)$. Then, the mean is modeled by GLM binomial regression with a logit-link, $logit\;\left(\mu_{ij}\right)=\beta_{j.}{\left(1,z_{i}\right)}^{⊤}$ where $\beta_{j.}=\left(\beta_{j0},\beta_{j1}\right)$ are regression coefficients for the intercept and phenotype. The model parameters are estimated using maximum likelihood estimation *via* the R package GAMLSS. Testing for differential abundance here is the equivalent of testing $H_{0}:\beta_{j1}=0$. ZIBSeq computes *q*-values for multiple testing from either a Chi-square or *t*-distribution depending on sample size.

The relative abundances are modeled by ZIBSeq one feature at a time; whereas, the ZIGDM models the abundances directly under a multivariate setting which can better capture the compositional effects. Further, the Dirichlet-multinomial (DM) distribution can account for overdispersion. But, the ZIGDM is a more flexible model than the DM. The novelty of the ZIGDM is two-fold: the use of the GDM to model count data and account for zero inflation with additional parameters had not been done before. The ZIGDM models the counts of each sample as $y_{i}.\sim ZIGDM\;\left(\omega_{i.},a_{i},b_{i.}\right)$ where $\pi_{ij}$ is a Bernoulli parameter that controls the absence probability, and $a_{ij}$ and $b_{ij}$ are beta distribution parameters that control the presence of feature j in sample i. The beta mean of the proportion of feature j in sample i is $a_{ij}/\left(a_{ij}+b_{ij}\right)$. By letting the dispersion parameter be $\sigma_{ij}=1/\left(1+a_{ij}+b_{ij}\right)$, the beta variance can be expressed as $\mu_{ij}(1-\left.\mu_{ij}\right)\sigma_{ij}$ which accounts for overdispersion. Similar to ZIBSeq, the ZIGDM also uses GLMs to model the mean parameter $\mu_{ij}$, but then uses score statistics and permutation p-values to test for differential abundance. The mean model is written as $logit\;\left(\mu_{ij}\right)=\beta_{j.}{\left(1,z_{i},x_{i.}\right)}^{⊤}$ where $\beta_{j.}=\left(\beta_{j0},\beta_{j1},\ldots ,\beta_{j,q+2}\right)$ are the regression coefficients for the intercept, phenotype, and covariates, respectively. Testing for differential abundance here is the equivalent of testing $H_{0}:\beta_{j1}=0$.

The beta-binomial distribution is the candidate for ℳ in corncob. In particular, corncob models the abundances as $y_{ij}\sim Binomial\;\left(N_{i},{\tilde{y}}_{ij}\right)$ to account for library size and relative abundance. Further, corncob assumes a beta prior on the probability parameter, which is expressed as ${\tilde{y}}_{ij}\sim Beta\;\left(a_{1j},a_{2j}\right)$, for model flexibility. Under this setting, the expected relative abundance of feature j in sample i is estimated by $\mu_{ij}=E\left({\tilde{y}}_{ij}\right)=a_{1j}/\left(a_{1j}+a_{2j}\right)$ and provides convenient support on (0,1) for modeling compositions *via* binomial regression. The binomial variance of $y_{ij}|N_{i}$ is $N_{i}\mu_{ij}\left(1-\mu_{ij}\right)\times \left[1+\phi_{ij}\left(N_{i}-\right.\right.1)]$ with an inflation factor of $1+\phi_{ij}\left(N_{i}-1\right)$ where $\phi_{ij}=1/\left(1+a_{1j}+a_{2j}\right)$. The flexibility of the prior allows corncob to account for library size and overdispersion when $\phi_{ij}$ is large. The mean model is written as $logit\;\left(\mu_{ij}\right)=\beta_{j.}{\left(1,z_{i},x_{i}\right)}^{⊤}$ and fitted using the trust region optimization algorithm for more efficient computation. The regression coefficients are given by $\beta_{j.}=\left(\beta_{j0},\beta_{j1},\ldots ,\beta_{j,q+2}\right)$ for the intercept, phenotype, and covariates, respectively. Testing for differential abundance here is the equivalent of testing $H_{0}:\beta_{j1}=0$. The parametric bootstrap Wald test is used to test for differential abundance.

ANCOM uses standard ANOVA to model the ALR-transformed relative abundances. ALR is based on Aitchison’s methodology of log ratios of compositional data [54]. The ALR transformation overcomes the unit-sum constraint on the relative abundances [18] and creates a map from the simplex S to R, which then allows for the use of classical statistical methods such as ANOVA [20]. Each feature is used as a reference feature one at a time which produces p(p-1) regression models [20]. Each model is written as

(3) $$\text{log}\frac{{\tilde{y}}_{ijg}}{{\tilde{y}}_{ij^{\prime }g}}=\alpha_{ij^{\prime }}+\beta_{jj^{\prime }g}+\epsilon_{ijj^{\prime }g}$$

where $\alpha_{jj^{\prime }}$ is the mean, $\beta_{jj^{\prime }g}$ captures the phenotypic group effect, and $\epsilon_{ijj^{\prime }}g$ is the error term for sample i, taxa $j\neq j^{\prime }$, and phenotype $z_{i}=g$. Covariates can also be included in the linear model when applicable. Testing for differential abundance is the equivalent of testing $H_{0}:\beta_{jj^{\prime }1}=\cdots =\beta_{jj^{\prime }G}$ using classical tests such as ANOVA, *t*-test, Wilcoxon, or Kruskal-Wallis depending on the number of groups and linear assumptions. The *p*-values are adjusted using the Benjamini–Hochberg procedure for multiple testing.

The above models are univariate with the exception of the ZIGDM. Multivariate models for analyzing microbiome data are also available. However, the multivariate methods do not perform better than the univariate methods for differential abundance analysis [43, 55]. An advantage of multivariate methods are the useful plots and numerical summaries of dimension reduction techniques including, but not limited to, principal components analysis (PCA), principal coordinates analysis (PCoA), canonical correlation analysis (CCA), and partial least squares discriminant analysis (PLS-DA) [43, 56]. For example, mixMC [43] is a multivariate method available in the mixOmics package in Bioconductor. Using CLR-transformed compositions, mixMC applies PCA to visualize diversity patterns and multivariate regression using sparse PLS-DA *via* a lasso penalty to select the most differentially abundant features. Lastly, mixMC also provides a multivariate approach for longitudinal differential abundance analysis.

The literature for longitudinal differential abundance analysis had been lacking until recently. The cost for sequencing has decreased over time allowing for the production of more longitudinal data [46]. Some earlier methods include Next maSigPro (microarray Significant Profiles) available in Bioconductor [44] and a negative binomial mixed effects model (NBME) available in the R package timeSeq [45]. Both Next maSigPro (the updated version of maSigPro) and NBME do not have normalization methods built into the software and so the user must normalize the data beforehand. Three methods developed around the same time for longitudinal differential abundance analysis include MetaSplines [46], MetaDprof [47], and MetaLonDA (Metagenomic Longitudinal Differential Abundance) [48, 57]. MetaSplines is available in metagenomeSeq, MetaDprof has no R package available, and MetaLonDA can be accessed through CRAN. MetaSplines, MetaDprof, and MetaLonDA apply a semi-parametric method known as smoothing spline ANOVA or SS-ANOVA to detect longitudinal differential abundance. MetaLonDA models the count data using the negative binomial distribution; whereas, MetaSplines and MetaDprof use the Gaussian distribution. MetaLonDA is designed to handle inconsistencies in time points, different number of samples per subject, and different number of subjects per phenotypic group. Further information regarding MetaSplines, MetaDprof, and MetaLonDA is provided in detail by [58]. More recently, NBZIMM [49] allows for the implementation of a negative binomial mixed effects model, zero-inflated negative binomial model, and Gaussian mixed effects model. NBZIMM is available for users in R *via* GitHub.

All of the above methods are useful. Appropriate model selection should be determined by a statistical procedure rather than by user choice. For example, a statistical procedure for identifying zero-inflated and hurdle distributions (iZID) was recently developed to appropriately model MSS data [59] and can be implemented in R *via* the iZID package [60]. Hurdle models, introduced by [61], are also referred to as zero-altered (ZA) models. Generally, ZA consists of one process that generates zeros and a second process truncated at zero that generates positive counts [62]. Unlike zero-inflated models, the slab of a zero-altered model cannot generate zeros. Wang et al. [60] provide the details of multiple zero-inflated and hurdle models along with any existing R packages. Keep in mind that there are other zero-inflated models not discussed in this paper that are available for microbiome data analysis. For example, a recent zero-inflated negative binomial model with a Dirichlet-process prior (ZINB-DPP) offers a Bayesian approach for differential abundance analysis [63].

The count data for edgeR and DESeq2 were sequenced *via* RNA-Seq. Other methods in this section such as metagenomeSeq and ZIBSeq generated the count data *via* 16S rRNA sequencing technology. An implication here is that these models were built for specific types of count data. Consequently, one must also consider the type of data when choosing a model. Further, corncob used soil microbiome data with three treatments; where as, human microbiome data were analyzed by the other methods. Of course, a zero-inflated model should be preferred when sparsity in the count data is high. Compared to metagenomeSeq, ZIBSeq is better suited for larger sample sizes and sparse count data based on its reported area under the receiver operating characteristic curve (AUC) *via* simulations. For smaller sample sizes, ZIBSeq is well-suited for multinomial and binomial data but not for zero-inflated Poisson or zero-inflated negative binomial data. Similar to ZIBSeq, corncob uses a single-feature modeling approach which does not take the compositional nature of the count data into consideration. So, a multivariate version of both of these two methods ought to be considered. Interestingly, ANCOM outperformed metagenomeSeq by significantly reducing FDR and increasing power even though it is not a zero-inflated model. However, ANCOM accounts for the compositionality of the count data using ALR normalization. ANCOM can also perform longitudinal analysis to test differential abundance at different time points. Later in 2020, Lin and Peddada [20] released ANCOM-BC which is an improved version of ANCOM that includes bias correction (BC). Most of the models above assume that abundance dispersions between groups are homogeneous. Both edgeR and DESeq2 use shrinkage to estimate dispersion and log-fold changes. Shrinkage helped to improve consistency and interpretation of results. The method of shrinkage is what sets edgeR and DESeq2 apart. Shrinkage in edgeR is determined by a user-adjusted parameter that depends on the prior degrees of freedom to impose weight on individual gene estimates and dispersions, which creates a weighted likelihood conditional on the count data. The conditional likelihood is based on the assumption that features with similar observed abundances have similar variances. The assumption of homogeneity may be problematic since phenotype level abundances can be influenced by multiple factors (e.g., other features, covariates, host, environment, etc.). The ZIGDM and corncob take differential dispersion into account, which is the assumption that dispersions between phenotype groups are heterogeneous. Also, tests for differential dispersion are available for the ZIGDM and corncob to determine if there is a significant association between dispersion and covariates. Notably, corncob was the first method to develop a test for differential dispersion. Finally, these models can be adjusted to include covariates. The ability to incorporate covariates into a model brings us to integrative analysis in the next section.

## 3. INTEGRATIVE ANALYSIS

There is an association between the microbiome and covariates including but not limited to metabolites, antibiotic usage, environmental factors, and host genetics that can influence host health [64, 65]. Recently, numerous associations between dietary covariates and taxa were implicated in the development of chronic diseases such as obesity [66]. The goal of integrative analysis is to identify and quantify associations between the microbiome and covariates. We discuss four methods for integrative analysis in this section including Dirichlet-multinomial regression or DMR [67], Dirichlet-multinomial Bayesian variable selection or DMBVS [68], a Bayesian zero-inflated negative binomial (ZINB) also referred to as IntegrativeBayes [19], and a Dirichlet-multinomial linear model with Bayesian variable selection or DMLMbvs [66]. Table 6 provides a summary of the methods discussed in this section and Table 7 summarizes the implementation of these methods in R.

The common biological motivation of each method is to determine if associations exist between any of the p features from $Y_{n\times p}$ and q covariates from $X_{n\times q}$ while controlling for the phenotypic response $z_{n\times 1}$. Statistical motivations here are similar to the differential abundance methods. DMR and DMLMbvs model the count data directly to account for issues arising from compositionality as well as overdispersion. DMBVS was highly interested in the connection between disease development and the association of the microbiome with other covariates. The lack of available models for integrative analysis motivated IntegrativeBayes to construct a model that could account for zero inflation and overdispersion.

Three of the models employ the DM, which is one of the distributions useful for model-based normalization. Other techniques of normalization decreases the power of the DM due to some loss of variation when using compositions. Thus, using the count data directly in the DM instead of the compositions results in better model performance [67]. The ZINB is most robust if the counts are first normalized using CSS when compared to other normalization methods. Further, the results of metagenomeSeq showed that CSS is highly beneficial for analyzing zero-inflated count data.

The first step of integrative analysis is to model the count data using Equation (1). The DM is the candidate for ℳ to denoise the count data in DMR, DMBVS, and DMLMbvs. The model learns the compositionality while also accounting for uncertainty and overdispersion in the count data. The count data are modeled as $y_{i}|\alpha_{i}.\sim DM\;\left(\alpha_{i}.\right)$ where $\alpha_{i}.=\left(\alpha_{i1},\ldots ,\alpha_{ip}\right)$ is the i-th sample row vector of normalized abundances estimated by the model. The DM parameter is strictly positive where each $\alpha_{ij}\in R^{+}$ and the unit-sum constraint has been removed.

The DM is derived by each model as follows [66–68]. The counts are assumed to follow a multinomial (Multi) distribution $y_{i}|N_{i},\psi_{i}\sim Multi\;\left(N_{i},\psi_{i}\right)$. Then, a Dirichlet (Dir) prior is placed on the multinomial parameter $\psi_{i.}|\alpha_{i}.\;\sim Dir\;\left(\alpha_{i}.\right)$. The DM is the result of integrating out the multinomial parameter, $\psi_{i}$. which is expressed as $f_{DM}\left(y_{i}|\alpha_{i.}\right)=\int p\left(y_{i}|N_{i},\psi_{i.}\right)p\left(\psi_{i.}|\alpha_{i}\right)d\psi_{i}$. Integrating out this parameter makes the model more efficient by having one less parameter. The variance of the DM is $Var\;\left(y_{ij}\right)=\left(N_{i}+A_{i}\right)/\left(1+A_{i}\right)E\left(\psi_{ij}\right)\left[1-E\left(\psi_{ij}\right)\right]N_{i}$, where $A_{i}=\sum_{j=1}^{p}\;\alpha_{ij}$. The variance is inflated by a factor of $\left(N_{i}+A_{i}\right)/\left(1+A_{i}\right)$ relative to the variance of the multinomial distribution. As a result, the DM model accounts for overdispersion in the count data. Letting $A_{i}$ tend toward zero will result in large overdispersion. If $A_{i}\to \infty$, the DM model reduces to a multinomial model. DMBVS and DMLMbvs are Bayesian adaptations of the DM.

IntegrativeBayes employs the NB as the candidate for the zero-inflated ℳ from Equation (2). If $r_{ij}=0$ in Equation (2), then the ZINB models the count data and their uncertainty while accounting for sample-wise sequencing depth $s_{i}$ by reparameterizing each sample taxon-specific negative binomial mean as the product of $s_{i}$ and the CSS normalized abundances, which are expressed as $\alpha_{ij}$. Further, the model also introduces a latent binary vector $\gamma =\left(\gamma_{1},\ldots ,\gamma_{p}\right)$ where $\gamma_{j}=1$ indicates the j-th feature is differentially abundant among the G groups. A beta-Bernoulli prior is placed on each $\gamma_{j}$ to quantify the proportion of features that are believed to be discriminatory. Then, the ZINB conditional on $r_{ij}=0$ is expressed as

(4) $$y_{ij}|r_{ij}=0,\gamma_{j},s_{i},\alpha_{ijg},\alpha_{ij0}∼\left\{\begin{array}{ll} \text{NB}\left(s_{i}\alpha_{ij0},\phi_{j}\right) & \text{if}\gamma_{j}=0\\ \text{NB}\left(s_{i}\alpha_{ijg},\phi_{j}\right) & \text{if}\gamma_{j}=1,z_{i}=g \end{array}\right.$$

where $\phi_{j}$ is the feature-specific dispersion parameter with a Gamma prior. The parameter $\phi_{j}$ captures overdispersion in the same manner as edgeR and DESeq2 described earlier.

Next, log-linear regression is employed by all four methods to identify any feature-covariate associations. The general framework of log-linear regression can be expressed as

(5) $$log\;\alpha_{ijg}=\beta_{j.}{\left(1,x_{i\cdot }\right)}^{⊤}$$

where the abundance-related response is $log\;\alpha_{ijg}$ for the covariates in sample i and group g. The feature-covariate regression coefficients are $\beta_{j}.\;=\left(\beta_{j0},\beta_{j1},\ldots ,\beta_{jq}\right)$ where the feature-specific intercept term is $\beta_{j0}$ and $\beta_{j1},\ldots ,\beta_{jq}$ estimate the associations between the j-th feature and the q covariates in the i-th sample. DMR, DMBVS, and DMLMbvs do not de*via*te from this specification. However, IntegrativeBayes expresses their log-linear regression model as

(6) $$\left\{\begin{array}{ll} \text{log}\;\alpha_{ij0}=\mu_{0j}+x_{i.}^{⊤}\beta_{j}. & \text{if}\;\gamma_{j}=0\\ \text{log}\;\alpha_{ijg}=\mu_{0j}+\mu_{gj}+x_{i\cdot }^{⊤}\beta_{j}. & \text{if}\;\gamma_{j}=1,z_{i}=g \end{array}\right.$$

where $\mu_{0j}$ is the feature-specific intercept term or baseline and $\mu_{gj}$ captures the baseline shift between the g-th group and reference group. Both $\mu_{0j}$ and $\mu_{gj}$ are assigned zero-mean Gaussian priors. The regression coefficients $\beta_{j}.\;=\left(\beta_{j1},\ldots ,\beta_{jq}\right)$ estimate the associations between the j-th feature and the q covariates in the i-th sample. Equations (5) and (6) do not require error terms because the uncertainty in the count data is accounted for by Equation (1) before regression is applied. DMLMbvs further calculates the mathematical balances $B\left(\psi_{i}.\right)$ of the taxa proportions $\psi_{i}$. estimated by ℳ to predict a continuous phenotypic response *via*

(7) $$z_{i}={\left(1,B(\psi {)}_{i.}\right)}^{⊤}\beta_{m}+\epsilon_{i}$$

where $\beta_{m}=\left(\beta_{0},\beta_{1},\ldots ,\beta_{M}\right)$ contains the regression coefficients for the balances. Further, $\beta_{0}$ is the intercept term with a zero-mean Gaussian prior, $\beta_{m}$ is the coefficient of balance m or $B(\psi {)}_{im}$, and the error term $\epsilon_{i}$ has a zero-mean Gaussian prior. Equation (7) requires an error term since the uncertainty in the phenotypic response is not accounted for in any previous steps. The multinomial prior $\psi_{i}$. estimates the compositions and is usually integrated out for model efficiency; however, DMLMbvs retains this parameter because the phenotypic response depends on the estimated compositions in Equation (7). Mathematical balances are constructed *via* random sequential binary partitioning of ψ, which results in a total of M=p-1 partitions. Mathematical balances help to identify a subset of features having the greatest association with the response rather than using a single feature selection approach.

Equations (5), (6), and (7) are high-dimensional with q×(p+1),q×(p+2), and p parameters, respectively. Multiple testing results in a loss of power in a high-dimensional setting and so regularization would help to increase the power [67]. Thus, regularization of Equations (5), (6), and (7) is necessary to optimize the model by reducing the parameter space [19]. Regularization for DMR, the only frequentist method here, includes both group and individual $\ell_{1}$ penalties and is optimized using an efficient block-coordinate descent algorithm, which utilizes a quadratic approximation of the log-likelihood function. Sparse $\ell_{1}$ regularization encourages sparsity in the regression coefficients, which is useful for identifying significant tax-acovariate associations whose regression coefficients are non-zero. Then, DMR uses the likelihood ratio test to identify significant feature-covariate associations. DMBVS, DMLMbvs, and IntegrativeBayes employ spike-and-slab priors to reduce the parameter space and for identifying significant feature-covariate associations. Spike-and-slab priors are conventional for Bayesian variable selection [19, 66, 68] and are defined as

(8) $$\beta_{jk}\sim \left(1-\delta_{jk}\right)I\left(\beta_{jk}=0\right)+\delta_{jk}N\left(0,\sigma_{\beta }^{2}\right)$$

where $\delta_{jk}=1$ indicates that feature j and covariate k are associated (i.e., $\beta_{jk}\neq 0$) and $\delta_{jk}=0$ otherwise. A beta-binomial prior is imposed on the latent binary variable $\delta_{jk}$ to control the number of significant associations selected by the model. The variance term $\sigma_{\beta }^{2}$ is assigned a conjugate prior (inverse-gamma) for model efficiency. Model fitting and sampling of non-zero regression coefficients is implemented using the Markov Chain Monte Carlo (MCMC) Metropolis-Hastings algorithm within a Gibbs sampler. The proportion of posterior samples with non-zero coefficients, or where $\delta_{jk}=\;1$, is called the posterior probability of inclusion (PPI) and makes a parsimonious quantification of uncertainty in variable selection. PPI above a certain threshold indicates the significance of any feature-covariate association, which is equivalent to testing $H_{0}:\beta_{jk}=0$. The null hypothesis is retained if PPI is below the chosen threshold and otherwise rejected. IntegrativeBayes uses a threshold that controls FDR; whereas, DMBVS and DMLMbvs use median PPI (i.e., 0.5) as the threshold.

Each of the four models here included numerous covariates. For instance, DMR included dietary intake of nutrients. DMBVS and IntegrativeBayes included molecular function covariates from the Kyoto Encyclopedia of Genes and Genomes (KEGG). IntegrativeBayes focused primarily on metabolomic covariates. DMLMbvs included multiple dietary covariates. The three DM-based models also included 16S rRNA count data as the features; whereas, IntegrativeBayes applied their model to MSS count data. IntegrativeBayes is the only model presented here that accounts for zero inflation in the count data, estimates the effect size for the discriminating features, and identifies differentially abundant features while simultaneously quantifying feature-covariate associations. Because IntegrativeBayes accounts for zero inflation, it is a well-suited model for MSS data. However, the ZINB is not the only zero-inflated model available for integrative analysis. A comprehensive review of other practical zero-inflated models is provided by [60, 74, 75]. All four models here account for overdispersion, compositionality, and high dimensionality of the data. DMLMbvs was the only model that had a continuous phenotype (body mass index or BMI); however, the authors noted that the model can be adjusted to include a categorical response. DMLMbvs uses the estimated compositional data to simultaneously identify feature-covariate associations and predict a continuous phenotypic outcome. The Bayesian approach is used by three of the four methods because it has several advantages over frequentist methods. Bayesian models can incorporate prior knowledge, quantify the uncertainty of model parameters, offer efficient model fitting *via* the MCMC algorithm, and calculate parsimonious inferential summaries such as PPI in variable selection.

## 4. NETWORK ANALYSIS

Microbial ecological interactions affect microbiome function and host health *via* the formation of complex communities with various symbiotic relationships where microbes coexist. Findings of a study implicated pH as a main factor for the networking of microbial communities in arctic soil [76]. The soil study found a two-cluster microbial network where one cluster was correlated to pH and the second was uncorrelated to pH. The goal of network analysis is to construct microbiome networks that characterize microbial ecological associations (i.e., taxa-taxa dependencies), which may help discover fundamental properties and mechanisms of microbial ecosystems [73]. Graphical models consist of nodes and edges, which are used to visualize the estimated microbial network. Each node corresponds to a taxon and an existing edge represents a direct association between any two nodes. Current statistical methods for network analysis estimate the correlation or partial correlation structure of the normalized count data to construct a network of nodes and edges [73]. Correlation-based methods include SparCC (Sparse Correlations for Compositional data) [69], CCLasso (Correlation inference for Compositional data through Lasso) [70], and REBACCA (Regularized Estimation of the BAsis Covariance based on Compositional dAta) [71]. Partial correlation-based methods include SpiecEasi (SParse InversE Covariance Estimation for Ecological Association Inference) [72] and HARMONIES (Hybrid Approach foR MicrobiOme Network Inferences *via* Exploiting Sparsity) [73]. SPRING (Semi-Parametric Rank-based approach for INference in Graphical model) [7] employs both correlation and partial correlation methods under a semi-parametric setting. Semi-parametric rank (SPR) correlation can be used as an alternative to correlation measures such as Pearson or Spearman, can be extended to partial correlation, and can account for zero inflation [7]. Table 8 provides a summary of the methods discussed in this section and Table 9 summarizes the implementation of these methods in R.

The main biological motivation of each of the above methods is the exigency to estimate correlation or partial correlation networks using normalized count data to make inferences about microbial interactions. Since the dawn of high-throughput next-generation sequencing technology, we have had the quantitative ability to characterize microbial communities [71] and learn how they associate with environmental conditions such as host health, metabolism, etc. [72]. The number of spurious taxa-taxa associations tend to be about three times the number of true associations and miss about 60% of the true associations when using conventional methods such as Pearson or Spearman correlation on compositional data [69]. Small sample sizes are common in microbiome studies, which can result in lower power for network inference [72]. Many taxa-taxa associations have not been verified in the literature and so benchmarking tools to assess model quality are needed [7, 73]. Statistical motivations are no different here than in previous sections. Microbiome data tend to be zero-inflated [7]. Further, microbiome data have high dimensionality, overdispersion, and sample heterogeneity [73]. Thus, network analysis models that consider the characteristics of microbiome data are most appropriate.

One of the main issues of microbiome data is the unit-sum constraint of compositional data. As stated earlier, Aitchison’s log-ratios are a useful normalization technique for compositional data. Methods such as SparCC, CCLasso, and REBACCA apply ALR normalization. SpieceEasi and SPRING apply CLR normalization. Unlike ALR, the CLR-transformed compositions are *p*-dimensional since no feature is used as a baseline. Both ALR and CRL map compositions from S to R. However, ALR can be more advantageous because these ratios are equivalent to the ratio of absolute abundances and have the subcompositional coherence property where the ratio of two features’ compositions is independent of other features [69]. HARMONIES applies model-based normalization where the count data are modeled directly *via* the parameters of the ZINB, which account for zero inflation, sample heterogeneity, overdispersion, and high dimensionality.

The methods discussed in this section use different approaches to estimate networks. Without loss of generality, we write

(9) $$\rho_{jj^{\prime }}\sim ℒ(\cdot )$$

where ℒ(⋅) is some process that estimates the covariance structure of the data, which is expressed as $\tilde{\Sigma }$, to infer the correlation $\rho_{jj^{\prime }}$ between features j and $j^{\prime }$. Depending on the transformation, the process estimates either a log-basis covariance (e.g., models that use ALR or CLR transformations) or model-basis covariance (e.g., probabilistic models such as ZINB). The various candidates for ℒ(⋅) used by each method are discussed below.

SparCC makes an iterative estimation of the correlation matrix based on the Aitchison log-ratio transformation of relative abundances of any two features with the assumption of sparsity, which can be loosely summarized in two steps. First, SparCC estimates the correlation matrix of the log-ratio transformation. Secondly through iteration, the pair with the greatest correlation is removed and the correlations are estimated again until certain criteria are met. The log transformation contains information of the true absolute abundances, or basis abundances, which means that the ratio of the relative abundances of any two features is equal to the ratio of their two basis abundances. Further, the ratio of any two relative abundances is independent of any ratio of other features. The relative abundances are estimated under a Bayesian framework where they are treated as being not fixed. While SparCC has advantages such as good overall performance, robustness to sparsity, and does not depend on any underlying distributions, it can be computationally intense and so it is not the most efficient model. Further, SparCC does not account for the influence of errors in the estimating equations that are used to estimate the correlation matrix (e.g., some of the estimated correlations can fall outside of [−1, 1]).

CCLasso accounts for the influence of error through the use of a loss function and for sparsity with an $\ell_{1}$ penalty term under a least squares framework to infer correlation structure. Similar to SparCC, CCLasso considers the compositional nature of microbiome data in estimating the correlation matrix with consistent accuracy. CCLasso has several advantages over SparCC. First, it produces a more accurate and positive definite correlation matrix. Second, all elements of the correlation matrix are contained in [−1, 1]. Third, CCLasso shrinks small correlations to zero, unlike SparCC, especially in the case of a shuffled microbiome dataset where correlations are actually zero.

REBACCA was published around the same time as CCLasso and so its performance was compared to only SparCC. While SparCC estimates the correlation matrix through an iterative procedure, REBACCA estimates the pairwise correlations by solving a linear system with deficient rank that is equivalent to the log-ratio transformations using $\ell_{1}$-norm shrinkage to induce sparsity in the network. The advantages of REBACCA include a more efficient algorithm, higher power, lower false positive rate (FPR), and more consistency than SparCC. Finally, it is worth noting that SparCC, CCLasso, and REBACCA are based on linear methods of correlation.

Partial correlation is a measure of association of two random variables after removing the effects of confounding variables. Controlling for confounding variables helps to reduce or eliminate spurious results. Further, partial correlation can be used to test for conditional independence of two random variables U and V given another random variable W [77]. Two features conditioned on abundances of all other features are conditionally independent if one of the two features provides no information about the abundance of the other feature. This implies that there is no direct association between the two features. The methods below estimate the partial correlation between features using several different approaches.

SPIEC-EASI employs two types of commonly used inference methods that utilize conditional independence for inferring sparse graphical models for CLR-transformed microbiome data. In short, covariance estimation and neighborhood selection are the two employed inference methods. Network sparsity is inferred through the Stability Approach to Regularization Selection (StARS), which uses subsampling to estimate a sparse network using minimal regularization [78]. For the first method, the graphical network is inferred through the estimation of the sparse inverse covariance matrix, which is regularized using graphical lasso (or glasso) so that indirect associations shrink to zero and only direct associations are selected. Glasso employs a penalized maximum likelihood approach with a global optimal solution for the reconstruction of the entire network. A Gaussian graphical framework is employed specifically because the inverse covariance matrix (or precision matrix) has the nice property of revealing conditionally dependent variables in the off-diagonal entries. For the second method, neighborhood selection is based on the known framework of Meinshausen and Bühlmann (MB method) to estimate local conditional independence one node at a time *via* Lasso [79]. Both inferential methods are advantageous because their formulations are convex optimizations, are useful for high-dimensional settings, can incorporate prior information regarding network topology, and outperformed SparCC overall.

SPRING is based on the use of novel SPR estimators of correlation and partial correlation for relative abundance data with a modified CLR transformation that can handle zero inflation. The modified transformation is rank-preserving and does not add a pseudo-value to zero counts. The semi-parametric model combines a truncated Gaussian copula graphical model with rank-based partial correlation to construct a sparse network using MB for neighborhood selection and StARS for model selection. SPRING outperformed existing methods such as SparCC for correlation inference and SPIEC-EASI for partial correlation inference. Also, the SPRING authors showed that Pearson correlation is not useful for identifying sparse partial correlations amongst features in microbiome data.

HARMONIES offers a competitive hybrid approach that includes a Bayesian zero-inflated negative binomial model with a Dirichlet process prior (ZINB-DPP). Further, glasso is applied to induce sparsity in the Gaussian graphical model that is regularized *via* StARS. The count data are normalized using a model-based approach *via* the DPP on the size factor $s_{i}$. ZINB-DPP accounts for overdispersion, zero inflation, sample heterogeneity, and high dimensionality making it a suitable model for estimating the true underlying abundances *via* the normalized abundances. Next, the posterior means of the normalized abundances on the log scale are used to fit a Gaussian graphical model to estimate the precision matrix for inferring the network with robust edge selection. HARMONIES demonstrated superior performance over existing methodologies including SPIEC-EASI and CCLasso in almost every scenario because it is designed to handle multiple challenges of microbiome data. Also, HARMONIES ensures proper biological interpretation of detected taxa-taxa associations by suggesting that all associated nodes are taxa of the same taxonomic level.

As an added bonus, SPIEC-EASI, SPRING, and HARMONIES each have their own novel synthetic data-generating tools that incorporate various network topologies to be used as a benchmark for assessing model quality. Synthetic data mimics real microbiome data and is currently a well-adapted way of assessing model quality due to the lack of a validated gold-standard network. MB-GAN (Microbiome Simulation *via* Generative Adversarial Network) [80] is another data synthesis tool useful for assessing model quality. MB-GAN addresses the challenges of simulating realistic microbiome data by learning from the given count data. The simulated count data are indistinguishable from the observed count data due to their similar properties such as sparsity, diversity, and taxa-taxa correlations. Other interesting features of MB-GAN include the use of real data as input without requiring model assumptions and efficient convergence.

Co-occurrence research within microbiomes has often focused on taxa-taxa associations. This is especially true in studies using amplicon sequencing techniques, such as 16S rRNA sequencing. However, recent work has begun to focus on reconstructing functional associations in metagenomic data [17, 81]. Genome-scale metabolic models (GEM), also known as Stoichiometric Metabolic Network models (SMN), computationally reconstruct and describe these associations [82]. The detailed information regarding the workflow, modeling, simulation, computational tools, and applications of GEMs can be found in [82–84]. These analyses used either the inferred or directly observed genetic content within the sampled metagenome to explore the microbial metabolic landscape. In addition, microbiome functional profiling of metagenomic data provides insight into what the microbial community has the potential to do at a molecular level [85]. With this in mind, research efforts have endeavored to model the community-scale metabolic potential encoded within metagenomes [83, 84]. These models not only need to consider the genetic content of the metagenome, but also must attempt to model fundamental arrangements of the microbial community, such as compartmentalization and availability of metabolites or nutrients, static or dynamic time constraints, and environmental sharing [84]. For example, Roume et al. [86] used a comparative multi-omic approach to reconstruct community-level metabolic networks within the microbial community present in wastewater. This type of analysis could aggregate the whole genetic content of the metagenome into one large community-scale organism. Taken together, metabolic modeling of functional metagenomic data allows for a closer mechanistic scrutinization of microbial co-occurrence within communities.

## 5. CONCLUSION AND OUTLOOK

In summary, multiple statistical methods for three major areas of microbiome research are available. Differential abundance analysis seeks to identify features that are discriminatory between phenotype groups. Since multiple diseases develop as a result of microbial dysbiosis, the results of differential abundance analysis may help find new and better ways to treat disease. Integrative analysis quantifies associations between taxa and covariates that potentially create an environment that enables the host to be more prone to disease. Understanding these associations between the microbiome and its environment can provide new insight to the cause, diagnosis and treatment of disease. Network analysis detects and quantifies taxa-taxa associations. Microbes commune with one another, which can modulate microbiome functions and host health. The methods discussed in this paper have been developed over the last decade due to the demand for statistical models that can handle the challenging characteristics of count data generated by high-throughput next-generation sequencing technology. At the very least, those challenges include zero inflation, overdispersion, sample heterogeneity, high dimensionality, correlation or partial correlation structure, technological variability, biological variability, normalization technique, and the compositional unit-sum constraint.

The available methods for analyzing microbiome data have greatly advanced metagenomic research. Great efforts have been made to account for the challenges of microbiome count data, to determine the normalization technique best suited for each model, and to assess model performance *via* available reference databases or synthetic data-generating tools. While some models do not account for the compositional nature of the data, it is suggested that this characteristic ought to be taken into consideration because the unit-sum constraint invalidates the assumption of independence of the data [87]. Future considerations should include methods for longitudinal studies, causal mediation analysis, and stochastic blocking, which are currently limited in the microbiome literature compared to other methods. Causal mediation analysis estimates the direct and indirect effects of predictor and mediating variables on the response variable [88]. An indirect effect is a relationship where there exists a pathway from a predictor variable to the response variable through a mediating variable; whereas, a direct effect is the relationship between only the predictor and response variables. For example, causal mediation could separate the effect of excessive alcohol consumption (predictor) on blood pressure (response) through a pathway such as BMI (mediator) [89]. The stochastic block model is an extension of network analysis where unsupervised learning is used to cluster the nodes of a network based on similar connectivity patterns [90, 91]. Unsupervised clustering methods can infer the number of clusters (or communities) that make up an entire network and the structure of taxa-taxa interactions within each community, which would then require scientific interpretation. Lastly, genomic reference databases need to be improved and updated because they are inadequate for the current needs of metagenomic research [92].

Microbiome research will continue to expand and present many complex statistical, scientific, and computing challenges. Future research must address these challenges in a collaborative effort of experts in statistics, science, and technology while building on the ideas from previous peer-reviewed research to offer reliable and interpretable solutions to the many important quests of microbiome research.

## Figures and Tables

**TABLE 1 |:** The notations and descriptions of typical microbiome data.

Label	Notation	Description
Count data	$Y_{n\;\times \;p}$	A *n* × *p* matrix of count data where each element $y_{ij}\in \mathbb{N}$ is the abundance for sample *i* = 1,…, *n* and feature *j* = 1,…, *p*. Denote $y_{i.}=\left(y_{i1},\ldots ,y_{ip}\right)$ as the 1 × *p* row vector of counts across all *p* features in sample i. Also, denote $y_{.j}={\left(y_{1j},\ldots ,y_{nj}\right)}^{⊤}$ as the *n* × 1 column vector of counts for feature *j* in all *n* samples. Denote ${\tilde{y}}_{ij}$ as relative abundance, ${\overset{⌣}{y}}_{ij}={\text{log}}_{2}\left(y_{ij}+c\right)$ as a log-transformed count with an added pseudo-value *c*, and library size $N_{i}=\sum_{j=1}^{p}\;y_{ij}$.
Covariates	$X_{n\;\times \;q}$	A *n* × *q* matrix where each element $x_{ik}\in \mathbb{R}$ is a measure for covariate *k* = 1,…, *q* in sample *i*. Denote $x_{i.}=\left(x_{i1},\ldots ,x_{iq}\right)$ as the 1 × *q* row vector of covariate measures across all *q* covariates in sample *i*. Also, denote $x_{\cdot k}={\left(x_{1k},\ldots ,x_{nk}\right)}^{⊤}$ as the *n* × 1 column vector of measures for covariate *k* in all *n* samples.
Phenotype	$z_{n\;\times \;1}$	A *n* × 1 column vector for the phenotypic response, which is written as ${\left(z_{1},\ldots ,z_{n}\right)}^{⊤}$ where each element *z*_*i*_ is the phenotypic response for sample *i*. The response is categorical* where *z*_*i*_ = *g* indicates the phenotypic group of each sample for group *g* = 1,…,*G*.

* Some models may have a continuous phenotypic response where each $z_{i}\in R$.

**TABLE 2 |:** Classes and alphabetized methods of statistical analyses discussed in this paper.

Differential abundance	Longitudinal differential abundance	Integrative analysis	Network analysis
ANCOM	maSigPro	DMBVS	CCLasso
corncob	MetaDprof	DMLMbvs	HARMONIES
DESeq2	MetaLonDA	DMR	REBACCA
edgeR	MetaSplines	IntegrativeBayes	SparCC
metagenomeSeq	mixMC		SpiecEasi
mixMC	MetaLonDA		SPRING
ZIBSeq	NBMM		
ZIGDM	NBZIMM		
ZINB-DPP			

**TABLE 3 |:** Summary of methods for differential abundance analysis in microbiome studies.

Method	Model assumption	Normalization	References	Availability
edgeR*	Negative binomial	TMM	[42]	Bioconductor
metagenomeSeq	Zero-inflated normal or log-normal	CSS	[24]	Bioconductor
DESeq2*	Negative binomial	RLE	[39]	Bioconductor
ANCOM	ANOVA	ALR	[40]	GitHub
ZIBseq	Zero-inflated beta	TSS	[5]	CRAN
ZIGDM	Zero-inflated generalized Dirichlet-multinomial	None^†^	[6]	CRAN
corncob	Beta-binomial	None^†^	[41]	GitHub
mixMC	PCA/sPLS-DA^‡^	CSS/TSS+CLR	[43]	Bioconductor
maSigPro*	Generalized linear models	User specified^††^	[44]	Bioconductor
NBME*	Negative binomial mixed effects	User specified^††^	[45]	CRAN
MetaSplines	Gaussian + SS-ANOVA	CSS	[46]	Bioconductor
MetaDprof	Gaussian + SS-ANOVA	TMM	[47]	Online
MetaLonDA	Negative binomial + SS-ANOVA	TMM/CSS^‡‡^	[48]	CRAN
NBZIMM	Negative binomial or Gaussian mixed effects	See below**	[49]	GitHub

* Developed for RNA-Seq data analysis.

** For these zero-inflated models, the negative binomial mixed effects model can incorporate the counts directly so normalization is not required; whereas, the Gaussian mixed effects model requires the arcsine square root transformation of the compositional data.

† These models do not require data normalization.

†† These models require the user to normalize the data beforehand.

‡ Principal components analysis (PCA) and sparse partial least squares discriminant analysis (sPLS-DA).

‡‡ Median-of-ratios scaling factor is a third normalization technique available in MetaLonDA.

**TABLE 4 |:** Implementation in R for differential abundance analysis in microbiome studies.

Method	Implementation	Updated
edgeR	https://bioconductor.org/packages/release/bioc/html/edgeR.html	2021
metagenomeSeq	https://rdrr.io/bioc/metagenomeSeq/	2021
DESeq2	https://bioconductor.org/packages/release/bioc/html/DESeq2.html	2021
ANCOM	https://github.com/FrederickHuangLin/ANCOM	2020
ZIBseq	https://cran.r-project.org/web/packages/ZIBseq/index.html	2017
ZIGDM	https://www.rdocumentation.org/packages/miLineage/versions/2.1	2017
corncob	https://github.com/bryandmartin/corncob	2021
mixMC	https://www.bioconductor.org/packages/release/bioc/html/mixOmics.html	2022
maSigPro	https://www.bioconductor.org/packages/release/bioc/html/maSigPro.html	2021
NBME	https://cran.r-project.org/web/packages/timeSeq/index.html	2019
MetaSplines	https://rdrr.io/bioc/metagenomeSeq/	2019
MetaDprof	https://cals.arizona.edu/~anling/sbg/software.htm	2016
MetaLonDA	https://cran.r-project.org/web/packages/MetaLonDA/index.html	2020
NBZIMM	https://github.com/nyiuab/NBZIMM	2022

**TABLE 5 |:** Summary of the count data models that appear throughout this paper.

Method	Model	Additional information
edgeR	$y_{ij}|\cdot \sim \text{NB}\left(\lambda_{ij},\phi_{j}\right)$ *	The mean parameter $\lambda_{ij}=N_{i}{\tilde{y}}_{ij}$ accounts for variation in library size and relative abundance.
DESeq2	$y_{ij}|\cdot \sim \text{NB}\left(\lambda_{ij},\phi_{j}\right)$	$\lambda_{ij}=s_{i}q_{ij}$ where *s*_*i*_, is estimated by the median-of-ratios method; *q*_*ij*_ is proportional to the amount of feature-wise cDNA fragments in a sample.
metagenomeSeq	${\overset{⌣}{y}}_{ij}|\cdot \sim N\left(\mu_{j},\sigma_{j}^{2}\right)$ **	In this zero-inflated model, $\pi_{i}\left(C_{i}\right)$ is the probability that an observed count is zero and is estimated *via $\text{logit}\left(\pi_{i}\right)=\beta_{0}+\beta_{1}\text{log}C_{i}$*, where *C*_*i*_, is the normalized abundance *via* CSS, $\mu_{j}$ and $\sigma_{j}^{2}$ are feature-specific Gaussian mean and variance.
ANCOM	Not applicable	Uses standard ANOVA to model the ALR-transformed relative abundances.
ZIBSeq	${\tilde{y}}_{ij}|\cdot \sim \text{Beta}\left(\mu_{ij},\phi_{ij}\right)$ ^†^	In this zero-inflated model, $\pi_{i}$, is the probability that a relative abundance is zero, $\mu_{ij}$ is the mean and $\phi_{ij}$ is precision. This parametrized beta distribution has shape parameters $\mu_{ij}\phi_{ij}$ and $\left(1-\mu_{ij}\right)\phi_{ij}$.
ZIGDM	^$y_{ij}|\cdot \sim \text{GDM}\left(\omega_{i.},a_{i.},b_{i\cdot }\right)$‡^	In this zero-inflated model, $\pi_{ij}$ is the probability that a count is zero.
corncob	$y_{ij}|\cdot \sim \text{Binomial}\left(N_{i},{\tilde{y}}_{ij}\right)$ ^††^	The prior on ${\tilde{y}}_{ij}$ is $p\left({\tilde{y}}_{ij}\right)=\text{Beta}\left(a_{1j},a_{2j}\right)$^‡‡^ with expected value $\mu_{ij}=a_{1j}/\left(a_{1j}+a_{2j}\right)$, which allows the use of the logit function to model the mean of the compositions.
DMR, DMBVS, DMLMbvs	$y_{i.}|\cdot \sim \text{DM}\left(\alpha_{i.}\right)$ ***	This zero-inflated model depends on a single parameter, $\alpha_{i}$, which can be interpreted as the model-based normalized abundances of sample *i*. Each $\alpha_{ij}\in {\mathbb{R}}^{+}$.
IntegrativeBayes	$y_{ij}|\cdot \sim NB\left(\lambda_{ij},\phi_{j}\right)$	In this zero-inflated model, the mean parameter is $\lambda_{ij}=s_{i}\alpha_{ijg}$ where size factor *s*_*i*_, is sequencing depth and $\alpha_{ijg}$ is the CSS normalized abundance of feature *j* in sample *i* and phenotypic group *g*. The feature-wise dispersion parameter is $\phi_{j}$.

* The NB pmf in general is $f(y|\cdot )=\frac{\Gamma (y+\phi )}{y!\Gamma (\phi )}{\left(\frac{\phi }{\lambda +\phi }\right)}^{\phi }{\left(\frac{\lambda }{\lambda +\phi }\right)}^{y}$.

** The normal pdf in general is $f\left(y|\mu ,\sigma^{2}\right)=\frac{1}{\sqrt{2\pi \sigma^{2}}}exp\;\left(-\frac{1}{2\sigma^{2}}(y-\mu {)}^{2}\right)$.

† The beta pdf in general can be parametrized as $f(y|\mu ,\phi )=\frac{\Gamma (\phi )}{\Gamma (\mu \phi )\Gamma (1-\mu )\phi )}y^{\mu \phi -1}(1-y{)}^{(1-\mu )\phi -1}$.

† The GDM pmf in general can be written as.

†† The binomial pmf in general is f(y∣⋅)=Nypx(1-p)N-y.

‡‡ This beta pdf is non-parametrized and written generally as $f(y|\cdot )=\frac{\Gamma (\alpha +\beta )}{\Gamma (\alpha |\Gamma (\beta )}y^{\alpha -1}(1-y{)}^{\beta -1}$.

*** The DM pmf in general is $f\left(y_{i}||\alpha_{i}\cdot \right)=\frac{\Gamma \left(\sum_{j=1}^{p}\;y_{\text{ij}}+1\right)\Gamma \left(\sum_{j=1}^{p}\;\alpha_{ij}\right)}{\Gamma \left(\sum_{j=1}^{p}\;y_{\text{ij}}+\sum_{j=1}^{p}\;\alpha_{j}\right)}\prod_{j=1}^{p}\;\frac{\Gamma \left(y_{\text{ij}}+\alpha_{ij}\right)}{\Gamma \left(y_{\text{ij}}+1\right)\Gamma \left(\alpha_{ij}\right)}$.

**TABLE 6 |:** Summary of methods for integrative analysis in microbiome studies.

Method	Model assumption	Normalization	Data type	Covariate type	References
DMR	Dirichlet-multinomial	None*	16S rRNA	Nutrient intake	[67]
DMBVS	Dirichlet-multinomial	None*	16S rRNA	KEGG pathways	[68]
IntegrativeBayes	Zero-inflated negative binomial	CSS	MSS	KEGG pathways; Metabolomics	[19]
DMLMbvs	Dirichlet-multinomial	None*	16S rRNA	Dietary	[66]

* The Dirichlet-multinomial model does not require data normalization.

**TABLE 7 |:** Implementation of methods for integrative analysis in microbiome studies.

Method	Implementation	Updated
DMR	http://statgene.med.upenn.edu/software.html	2013
DMBVS	https://github.com/duncanwadsworth/dmbvs	2017
IntegrativeBayes	https://github.com/shuangj00/IntegrativeBayes	2019
DMLMbvs	https://github.com/mkoslovsky/DMLMbvs	2020

**TABLE 8 |:** Summary of methods for network analysis in microbiome studies.

Method	Network type	Method	Normalization	References	Application
SparCC	Correlation	Iterative estimation of correlation	ALR	[69]	GitHub
CCLasso	Correlation	Least squares with /_1_ penalty	ALR	[70]	GitHub
REBACCA	Correlation	Fast /_1_ - norm shrinkage	ALR	[71]	Online
SpiecEasi	Partial correlation	Gaussian graphical model	CLR	[72]	GitHub
SPRING	Partial correlation, SPR correlation	Truncated Gaussian copula model	Modified CLR	[7]	CRAN
HARMONIES	Partial correlation	Gaussian graphical model	DPP	[73]	GitHub

**TABLE 9 |:** Implementation of methods for network analysis in microbiome studies.

Method	Implementation	Updated
SparCC	https://www.rdocumentation.org/packages/SpiecEasi/versions/1.0.7/topics/sparcc	2012
CCLasso	https://github.com/huayingfang/CCLasso	2016
REBECCA	https://faculty.wcas.northwestern.edu/hji403/REBACCA.htm	2015
SpiecEasi	https://github.com/zdk123/SpiecEasi	2021
SPRING	https://rdrr.io/github/GraceYoon/SPRING/man/SPRING.html	2020
HARMONIES	https://github.com/shuangj00/HARMONIES	2020
